# Pancreaticojejunostomy For Severe Symptomatic Chronic Pancreatitis

**DOI:** 10.1155/1992/97549

**Published:** 1992

**Authors:** G. W. L. Denton, W. A. Brough, D. E. F. Tweedle

**Affiliations:** University Hospital of South Manchester Nell Lane Withington Manchester M20 8LR UK

## Abstract

The role of operation, particularly pancreaticojejunostomy, in the treatment of abdominal pain from chronic pancreatitis is controversial, but relief of pancreatic duct obstruction may decrease the rate of pancreatic organ failure. Our results over 6 years in 13 carefully selected patients suggest that pancreatic drainage does relieve pain but is less effective in preventing pancreatic exocrine failure. Pain was the indication for operation in all patients.


*HPB Surgery*, 1992, Vol. 5, pp. 117–122
Reprints available directly from the publisher
Photocopying permitted by license only

© Harwood Academic Publishers GmbH
Printed in the United Kingdom

### PANCREATICOJEJUNOSTOMY FOR SEVERE SYMPTOMATIC CHRONIC PANCREATITIS

G.W.L. DENTON, W.A. BROUGH and D.E.F. TWEEDLE
*University Hospital of South Manchester, Nell Lane, Withington, Manchester M20 8LR*

*(Received 7 August 1991)*

The role of operation, particularly pancreaticojejunostomy, in the treatment of abdominal pain from chronic pancreatitis is controversial, but relief of pancreatic duct obstruction may decrease the rate of pancreatic organ failure. Our results over 6 years in 13 carefully selected patients suggest that pancreatic drainage does relieve pain but is less effective in preventing pancreatic exocrine failure. Pain was the indication for operation in all patients.

KEY WORDS: Chronic pancreatitis, pancreaticojejunostomy, pancreatic exocrine function, pancreatic endocrine function

### INTRODUCTION

There may be many causes of pain in chronic pancreatitis[1], but surgeons have long held the view that increased pancreatic ductal pressure is one of them. Recently, ductal pressure has been shown to be greater in patients with painful chronic pancreatitis than in those in whom it was painless[2]. Patients with dilated pancreatic ducts can best be treated by pancreaticojejunostomy[3–13], either distal (end to end) pancreaticojejunostomy[3-5,11] or longitudinal pancreaticojejunostomy in those with multiple strictures of the duct of Wirsung.

Although there has been some indication in patients with dilated ducts that pancreaticojejunostomy may slow the progression of pancreatic failure[7], initial hopes that operation would improve pancreatic function have not been fulfilled. In this paper we present our recent experience of 13 carefully selected patients who underwent pancreaticojejunostomy for chronic pancreatitis.

### PATIENTS AND METHODS

The thirteen patients were selected from 211 patients referred to this unit with chronic pancreatitis during the six year period 1983–88. There were nine males and four females with a median age of 42 years (range 25–60). The clinical diagnosis of chronic pancreatitis was confirmed in all patients by endoscopic retrograde cholangiopancreatography (ERCP); additional confirmation was obtained in six patients

Address correspondence to: Mr. D.E.F. Tweedle, University Hospital of South Manchester, Nell Lane, Withington, Manchester M20 8LR

by computerised axial tomography. All patients had a dilated pancreatic duct on ERCP with a minimum diameter of 10mm and "marked changes of chronic pancreatitis" according to international definitions[14]. Five had minor degrees of parenchymal calcification and nine had stones in the pancreatic duct. Endocrine failure was defined as diabetes mellitus requiring either oral hypoglycaemic agents or insulin. Exocrine failure was defined as steatorrhoea in excess of 18mmol (5g) of fat per day and necessitating exogenous pancreatic enzymes. Excessive alcohol consumption was defined as consumption of more than 8 units per day (1 unit = 8g alcohol).

Five patients had undergone previous operation. Two had undergone cholecystectomy (one with exploration of the common bile duct) and two had undergone internal drainage procedures for pseudocysts. One had undergone distal pancreatectomy for chronic pancreatitis but was still in pain and had a dilated pancreatic duct in the pancreatic remnant. Four patients had pancreatic exocrine failure and three were diabetic preoperatively. In eleven patients the cause of the chronic pancreatitis was excessive alcohol consumption, but only one patient had not stopped drinking at the time of operation. All patients were strongly advised to refrain from consumption of alcohol for the rest of their lives and were counselled by the alcoholic dependency unit. In the other two patients the aetiology of their chronic pancreatitis was never established, although excessive consumption of alcohol was suspected.

The indication for operation was constant, severe abdominal pain that disrupted normal daily living, coupled with an apparent desire to refrain from alcohol consumption. All patients were consuming large quantities of oral analgesics. All but two required opiates to relieve their pain and four had undergone percutaneous coeliac ganglion blockade.

Pancreaticojejunostomy was the operation of choice when dilation of the main pancreatic duct was seen at ERCP in the absence of gross parenchymal calcification. These operations were performed by one surgeon (DEFT). Ten patients had the Partington-Rochelle procedure of longitudinal pancreaticojejunostomy[15]. Three patients had the Puestow-Gillesby procedure in which splenectomy and distal pancreatectomy are combined with longitudinal incision of the pancreatic duct, again with anastomosis to a Roux loop of jejunum[16]. In each case the incision in the pancreatic duct was made as long as possible (minimum 10 cm). In addition, three patients underwent biliary drainage (one cholecystojejunostomy, two choledochojejunostomies) and one patient underwent drainage of a pseudocyst.

### RESULTS

One patient died twelve hours after operation from disseminated intravascular coagulopathy. The recovery of three patients who were heavy smokers was complicated by pulmonary infections. One patient developed a left subphrenic abscess which required surgical drainage on two occasions but from which he made a full recovery. In one patient there was a leak from the pancreatic anastomosis which settled with conservative treatment. One other patient needed reoperation to drain a lymphatic collection in the lesser sac and ligate a lymphatic vessel after failed percutaneous drainage.

Of the twelve surviving patients, one (a surgeon) returned abroad, but has remained in contact. The other eleven had been reviewed every six months for a median period of 52 months (range 28–86). Complete relief of pain was achieved in nine patients. Some relief was achieved in two patients, one of whom continues to ingest alcohol. The last patient has had no pain relief but continues to consume excessive quantities of alcohol.

Exocrine pancreatic function has deteriorated since pancreaticojejunostomy in four patients, who now require enzyme supplements. By contrast, it has improved in another two patients who had preoperative steatorrhoea. Following the operation the one patient who still consumes alcohol regularly now requires insulin, and antoher patient who has been started on an oral hypoglycaemic agent. Thus exocrine function was unchanged in six of twelve survivors, while endocrine function was unchanged in ten. No patient has lost body weight during the period of postoperative follow up. Anastomotic patency was demonstrated by subsequent ERCP in four patients whose exocrine function deteriorated despite ceasing to drink alcohol.

### DISCUSSION

Chronic pancreatitis is usually a progressive disease characterised by chronic abdominal pain, exocrine pancreatic failure and diabetes mellitus. Operative treatment whether by duct drainage[3–13] or resection[17–19] is usually considered to improve abdominal pain, although this fact has been disputed[20]. Most clinicians have found that endocrine and exocrine function are not improved by operation, but one study has suggested that the progression of pancreatic failure may be slowed by pancreaticojejunostomy[7].

Drainage of the pancreatic duct by end to side anastomosis between the pancreatic duct and the jejunum following distal pancreatectomy and splenectomy was described in 1954 by Du Val[21]. This procedure was improved in 1958 by Puestow and Gillesby who made a longitudinal opening in the pancreatic duct for anastomosis to the jejunum[15]. In 1960 Partington and Rochelle performed a longitudinal side to side anastomosis between the pancreatic duct and a Roux loop of jejunum[16], thus avoiding the need for splenectomy and potential haemorrhage from dissecting the distal pancreas involved in a chronic inflammatory process. This modified Puestow operation is now the most commonly performed anastomosis.

On this unit, we have a very conservative approach to patients with chronic pancreatitis, patients undergo operation only after careful selection. During the six years under review, 211 patients with chronic pancreatitis were referred for assessment and only eight patients underwent pancreatic resection. The 13 patients who underwent pancreaticojejunostomy for intolerable pain were selected because they had dilated ducts without evidence of gross parenchymal calcification. In common with other studies, failure to relieve pain was seen in two patients who continue to drink alcohol, but nine of twelve survivors (75%) are free of pain up to 86 months after operation. Although these operations may have corrected exocrine insufficiency in two patients and prevented the progression of exocrine and endocrine failure in another five the length of follow up is short and further organ failure may become apparent with time (especially since ERCP has shown anastomotic patency).

Exocrine function has deteriorated in three of the seven patients with non-calcific disease but in only one of five with calcific disease (two of whom actually improved). These results reinforce the lack of any correlation between exocrine insufficciency and the presence of calcification[18]. By contrast, among patients without preoperative diabetes, two of three with calcific disease have become diabetic, but none of six with non-calcific disease.

Overall, these results are better than many previous reports and probably reflect the very careful selection of patients. We believed that all but one of the alcoholics had stopped drinking for at least one year, none were insulin dependent diabetics and none showed severe parenchymal calcification.

### *References*

1. Ihse, I.(1990) Pancreatic Pain. *Br. J. Surg.*, **77**, 121–122
2. Okazaki, K., Yamamoto, Y. and Kagiyama, S. *et al.* (1988) Pressure of papillary zone and pancreatic main duct in patients with chronic pancreatitis in the early state. *Scand. J. Gastroenterol.*, **23**, 501–506
3. Mannell, A., Adson, M.A., McIlrath, D.C. and Ilstrup, D.M. (1988) Surgical management of chronic pancreatitis: long term results in 141 patients. *Br. J. Surg.*, **75**, 467–472
4. Moosa, A.R. (1987) Surgical treatment of chronic pancreatitis: an overview. *Br. J. Surg.*, **74**, 661–667
5. Sato, T., Miyashita, E., Yamauchi, H. and Matsuno, S. (1986) The role of surgical treatment of chronic pancreatitis. *Ann. Surg.*, **203**, 266–271
6. Cooper, M.J. and Williamson, R.C.N. (1984) Drainage operations in chronic pancreatitis. *Br. J. Surg.*, **71**, 761–766
7. Nealon, W.H., Townsend, C.M. and Thompson, J.C. (1988) Operative drainage of the pancreatic duct delays functional impairment in patients with chronic pancreatitis. *Ann. Surg.*, **208**, 321–326
8. Escallon, A. and Aldrete, J.S. (1986) Lateral pancreaticojejunostomy for pain relief in chronic pancreatitis: analysis of effectiveness in 19 patients. *South M.J.*, **79**, 936–940
9. Holmberg, T., Isaksson, G. and Ihse, I. (1985) Long term results of pancreaticojejunostomy in chronic pancreatitis. *Surg. Gynaecol. Obstet.*, **160**, 339–346
10. White, T.T. and Slavotinek, A.H. (1979) Results of surgical treatment of chronic pancreatitis: report of 142 cases. *Ann. Surg.*, **188**, 129–133
11. Prinz, R.A. and Greenlee, H.B. (1981) Pancreatic duct drainage in 100 patients with chronic pancreatitis. *Ann. Surg.*, **194**, 313–320
12. Warshaw, A.L. (1985) Conservation of pancreatic tissue by combined gastric, biliary and pancreatic duct drainage for pain from chronic pancreatitis. *Am. J. Surg.*, **149**, 563–569
13. Bradley, E.L. (1987) Long term results of pancreaticojejunostomy in patients with chronic pancreatitis. *Am. J. Surg.*, **153**, 207--213
14. Axon, A.T.R., Classen, M., Cotton, P.B., Cremer, M., Freeny, P.C. and Lees, W.R. (1984) Pancreatography in chronic pancreatitis: international definitions. *Gut*, **25**, 1107–1112
15. Puestow, C.B. and Gillesby, W.I. (1958) Retrograde surgical drainage of pancreas for chronic relapsing pancreatitis. *Arch. Surg.*, **76**, 898–904
16. Partington, P.F. and Rochelle, R.E.L. (1960) Modified Puestow procedure for retrograde drainage of the pancreatic duct. *Ann. Surg.*, **152**, 1037–1043
17. Cooper, M.J. and Williamson, R.C.N. (1987) Resection in chronic pancreatitis. *Br. J. Surg.*, **74**, 807–812
18. Lambert, M.A., Linehan, I.P. and Russell, R.C.G. (1987) Duodenum preserving total pancreatectomy for end stage pancreatitis. *Br. J. Surg.*, **74**, 35–39
19. Linehan, I.P., Lambert, M.A., Brown, D.C., Kurtz, A.B., Cotton, P.B. and Russell, R.C.G. (1988) Total pancreatectomy for chronic pancreatitis. *Gut*, **29**, 358–365
20. Ammann, R.W., Akovbiantz, A., Largiarder, F. and Schuler, G. (1984) Course and outcome of chronic pancreatitis. *Gastroenterology*, **86**, 820–828
21. DuVal, M.K. (1954) Caudal pancreaticojejunostomy for chronic pancreatitis. *Ann. Surg.*, **140**, 775–785

*(Accepted by S. Bengmark 27 August 1991)*

### INVITED COMMENTARY

This report of a small but carefully selected series of patients with chronic (alcoholic) pancreatitis confirms the value of longitudinal pancreaticojejunostomy for the control of pain. Follow-up ranged from two to seven years, when nine of twelve survivors were free of pain. Our own experience and that of others (as cited by the authors) has shown that provided the main pancreatic duct is grossly dilated, preferably greater than 10 mm in diameter, satisfactory analgesia can be anticipated in at least 70 per cent of patients following an adequate ductal drainage procedure. The long-side-to-side anastomosis of the Partington-Rochelle operation is much more likely to remain patent than the end-to-side lateral pancreaticojejunostomy originally devised by DuVal. In line with previous work[1] anastomotic patency was confirmed in all four patients studied with postoperative ERCP in the present series.

Progressive pancreatitis probably accounted for the three symptomatic failures (25 per cent) and the deterioration of pancreatic function in some of the other patients. Two of the three unsatisfactory results were in patients who continued to drink alcohol to excess, and the poor prognostic portent of persistent alcoholism is well known in this disease. Unfortunately pancreatitis can smoulder on even among those who stop drinking, though it is reasonable to hope that a timely drainage operation will slow down and sometimes stop the progression of the disease. Endocrine function would not be expected to alter as a result of pancreaticojejunostomy, nor should exocrine function improve unless there were a complete ductal stricture in the head of pancreas which can be shortcircuited into the gut. In our experience of "ambrigrade" operative ductography, however, instillation of contrast into an ectatic duct nearly always produces free flow into the duodenum[2]. Thus ductal strictures are seldom critically tight.

We believe that the absence of complete ductal stenosis in patients with a dilated system makes ductal hypertension an unconvincing explanation for pain in this type of chronic pancreatitis. We have recently confirmed the findings of Ebbehoj and colleagues[3] that pancreatic tissue pressure is elevated in this disease. Using fine needle cannulation of the pancreas at operation and a flow infusion system, we have found an increased parenchymal resistance to infusion in a group of 17 patients with chronic pancreatitis[4]. Mean values varied between about 150–250 mm Hg at different sites in the gland, compared with 20 mm Hg in a small reference group of patients with an essentially normal pancreas. Thus parenchymal hypertension may produce a compartment syndrome, and the effect of longitudinal pancreaticojejunostomy might be likened to that of a fasciotomy in the leg.

The particular advantage of pancreaticojejunostomy over a pancreatectomy is that it avoids the need to mobilise the pancreas from its bed (beside doing nothing to exacerbate pancreatic insufficiency). Three patients in the present series were submitted to the Puestow procedure in which the body of pancreas is "filleted" and invaginated into a Roux loop after resection of the pancreatic tail and spleen. Most surgeons have abandoned the Puestow operation nowadays because of the bloody dissection entailed, though we have occasionally combined distal hemipancreatectomy with Roux loop drainage of a dilated duct in the head of pancreas[5]. The authors had three or four serious complications, including one death from haemorrhage, among their 13 patients with pancreaticojejunostomy. It would certainly have been useful to know whether some of these problems stemmed from the Puestow operation.

The authors admit to taking a very conservative approach to patients with chronic pancreatitis. The present paper reveals them to be ultra-conservative, offering operation to a mere 10 per cent of patients (21 ex 211) with chronic pancreatitis seen over a six-year period. No doubt we have a different referral practice but our own rate for surgical intervention is very much higher, well over 50 per cent. Although careful selection is of paramount importance, operative treatment can generally achieve satisfactory results, and even total pancreatectomy is attended by a relatively low mortality rate (five per cent).

*References*

1. Warshaw, A.L., Popp, J.W. Jr and Schapiro, R.H. (1980) Long-term patency, pancreatic function, and pain relief after lateral pancreatico-jejunostomy for chronic pancreatitis. *Gastroenterology*, **79**, 289–293
2. Desa, L.A. and Williamson, R.C.N. (1990) On-table pancreatography: importance in planning operative strategy. *Br. J. Surg.*,. **77**, 1145–1150
3. Ebbehoj, N., Borly, L., Bulow, J., Rasmussen, S.A. and Madsen, P. (1990) Evaluation of pancreatic tissue fluid pressure and pain in chronic pancreatitis. A longitudinal study. *Scand. J, Gastroenterol.*, **25**, 462–466
4. Jalleh, R.P. Aslam, M. and Williamson, R.C.N. (1991) Pancreatic tissue and ductal pressures in chronic pancreatitis. *Br.J. Surg.*, (in press)
5. Aldridge, M.C. and Williamson, R.C.N. (1991) Distal pancreatectomy with and without splenectomy. *Br. J. Surg.*, (in press)
6. Cooper, M.J., Williamson, R.C.N. and Benjamin, I.S. *et al.* (1987) Total pancreatectomy for chronic pancreatitis. *Br. J. Surg.*, **74**, 912–915

R.C.N. Williamson
N. Bliouras
Department of Surgery
Royal Postgraduate Medical School
Hammersmith Hospital
DuCane Road
London W1 0NN, UK

